# Quantum Anomalous Hall Effect and Tunable Topological States in 3*d* Transition Metals Doped Silicene

**DOI:** 10.1038/srep02908

**Published:** 2013-10-09

**Authors:** Xiao-Long Zhang, Lan-Feng Liu, Wu-Ming Liu

**Affiliations:** 1 Beijing National Laboratory for Condensed Matter Physics, Institute of Physics, Chinese Academy of Sciences, Beijing 100190, China

## Abstract

Silicene is an intriguing 2D topological material which is closely analogous to graphene but with stronger spin orbit coupling effect and natural compatibility with current silicon-based electronics industry. Here we demonstrate that silicene decorated with certain 3*d* transition metals (Vanadium) can sustain a stable quantum anomalous Hall effect using both analytical model and first-principles Wannier interpolation. We also predict the quantum valley Hall effect and electrically tunable topological states could be realized in certain transition metal doped silicene where the energy band inversion occurs. Our findings provide new scheme for the realization of quantum anomalous Hall effect and platform for electrically controllable topological states which are highly desirable for future nanoelectronics and spintronics application.

The recently discovered topological insulators (TIs)^1^,^2^,^3^,^4^,^5^ have aroused great interest in the fields of condensed matter physics and materials science due to the metallic boundary states protected by time-reversal symmetry (TRS). TIs have also become perfect breeding ground for a variety of exotic quantum phenomena^1^,^2^. In particular, breaking the TRS respected by TIs via magnetic doping is predicted to give birth to the Majorana fermion^6^, topological magnetoelectric effect^7^, and the so-called quantum anomalous Hall effect (QAHE)^8^,^9^,^10^,^11^,^12^,^13^,^14^,^15^, which has quantised Hall conductance in the absence of external field^16^ and can be intuitively thought as half of TIs. Since TIs have been fabricated in materials ranging from 2D^17^ to 3D^18^, engineering these novel phenomena in real materials represents one of the most fascinating areas in this field.

As the 2D TI (also known as quantum spin Hall (QSH) insulator) graphene^3^,^4^,^19^ has been altering the research direction of nanoelectronics from silicon-based materials to carbon-based ones, however, the advent of silicene^20^,^21^,^22^,^23^,^24^, which is the silicon equivalent for graphene, seems to turn the tide. Silicene is closely analogous to graphene in the sense that it consists of a single layer of Si atoms arranged to a low buckled honeycomb lattice, and its low energy physics can be described by Dirac-type energy-momentum dispersion akin to that in graphene^20^, hence the inherited many intriguing properties, including the expected Dirac fermions and QSH effect^25^. Yet a striking difference between silicene and graphene is that the stable silicene monolayer has additional buckling degree^20^, which accounts for the relatively large (1.55 meV) spin orbit coupling (SOC) induced gap^25^ in silicene and a couple of unusual quantum phenomena recently reported^26^,^28^,^29^,^30^. Indeed, these features together with the natural compatibility with current silicon-based microelectronics industry make silicene a promising candidate for future nanoelectronics application. Moreover, from the view of practical applications, it is highly appreciated if magnetism or sizable band gap or both, like in QAHE with additional edge states protected by topology, can be established in the nonmagnetic silicene, especially in the presence of the bulking degree.

In this work, we explorer the underlying topological nontrivial states of silicene through a systematic investigation of adsorption of 3*d* transition metals (TM). We demonstrate that 3*d* TM strongly bonding with silicene and the TM-silicene systems are strongly magnetic. From combined tight-binging (TB) model analysis and first-principles Wannier interpolation, we show that the Vanadium doped silicene hosts a stable QAHE which survives strong correlation effect of the adatom, and this system can also be half-metallic^31^ if the Fermi level is properly tuned. Further, a close study of the TB model in the band inverted regime gives rise to another topologically nontrivial state, which supports quantum valley Hall effect (QVHE)^32^. We predict the resulting QAHE and QVHE can be tuned directly using an external electrical field, which is rather appealing for future nanoelectronics and spintronics application.

## Results

### Adsorption and magnetism analysis

**Figure 1:**
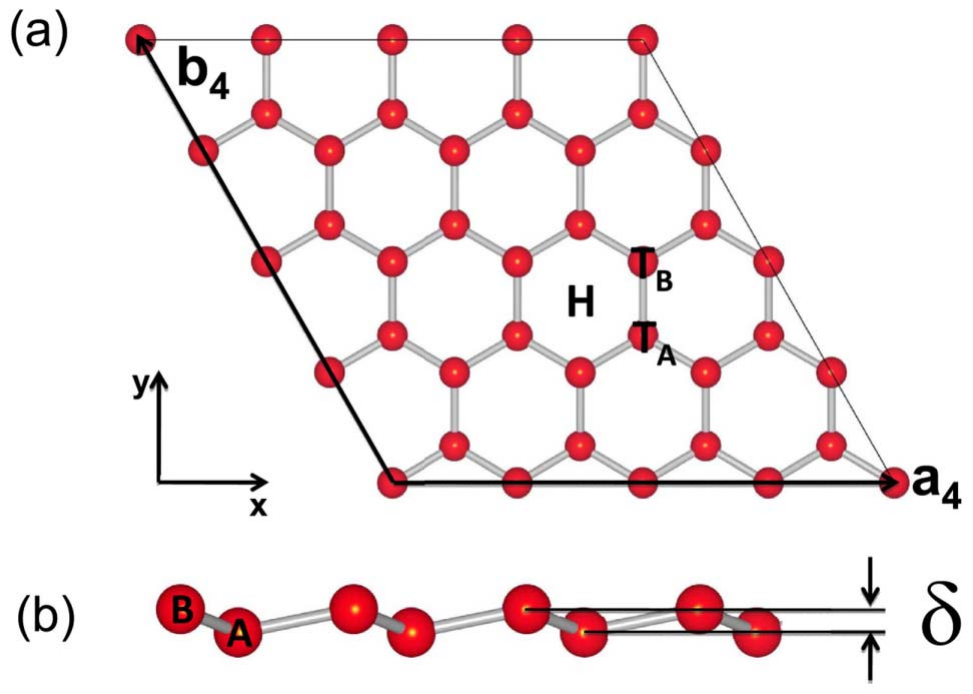
The lattice geometry of 3*d* transition metals doped silicene. The lattice geometry of 3*d* transition metals doped silicene with lattice constant |*a*_4_| = |*b*_4_| = 4*a*, where *a* = 3.86 *Å* is the lattice constant of silicene. (a) Top view of 4 × 4 silicene monolayer where the 3 adsorption sites (Hollow (H), top of A sublattice (T*_A_*) and B sublattice (T*_B_*)) are marked out with black letters. (b) Side view of silicene, the two equivalent Si sublattices are labeled as A and B, respectively, with a buckled distance *δ* = 0.44 *Å*.

We use 4 × 4 supercell of silicene to model the interaction between 3*d* TM (Sc, Ti, V, Cr, Mn, Fe, Co, Ni) and silicene. As silicene has buckled geometry, we consider three high symmetry adsorption sites, namely the hollow (H) site at the center of a hexagon, two top sites denoted as T*_A_* and T*_B_* corresponding to the top of Si atoms belonging to A and B sublattice, respectively (see Fig. 1). To evaluate the effect of on-site Coulomb interactions among 3*d* electrons of adatoms on the equilibrium structure and magnetic properties of the TM-silicene system, the simulations have been carried out within generalized gradient approximation (GGA)^34^ and GGA + *U*^35^,^36^ framework separately.

Let us first focus on the GGA case. From our first-principles results, of the three adsorption sites concerned, all 3*d* TM energetically favor H site, which is 0.02 eV ~ 0.60 eV and 0.20 eV ~ 0.80 eV higher in adsorption energy (Δ*E* = *E_s_* + *E_ads_* − *E_s–ads_* is the adsorption energy within GGA, where *E_s_*, *E_ads_*, *E_s–ads_* are energies of the 4 × 4 pristine silicene, single adatom, and silicene-adatom system, respectively.) than T*_A_* and T*_B_* site, respectively (see Supplementary Fig. S2). The bondings between 3*d* TM and silicene are strongly covalent as manifested by a much larger Δ*E* ranging from 2.44 eV to 4.75 eV. The unusual large Δ*E* compared with that in graphene case^12^,^37^ could be related to the covalently more active *sp*^3^-like orbitals of silicene, which result from the unique buckled geometry.

Much like the graphene case^12^,^37^, most of TM (except Ni) doped silicene exhibit magnetism with sizable magnetic moments ranging from ~1 *μ_B_* to ~5 *μ_B_*. A relatively large magnetic moment is key to the realization of QAHE in silicene which we will discuss later. We also note that when some TM (Sc, Ti, Cr) adsorbing on H site, the density of states (DOS) show peaks at the Fermi level, indicating that these systems could be magnetic instable and may undergo Jahn-Teller distortion to lower total energy. In the case of Sc-silicene, we artificially move one of three Si atoms nearest to Sc to break the *C*_3_ rotational symmetry. After relaxation the 3 nearest Si atoms to Sc which originally coplanarly arranged themselves as a regular triangle (the bond length *d_Si–Sc_* equal to 2.62 *Å*) now distort to a isosceles triangle (*d_Si–Sc_* become 2.63 *Å*, 2.63 *Å* and 3.12 *Å*) by pushing the moved Si atom down away from the upper sublattice plane by 1.24 *Å*. The distorted Sc-silicene system becomes more stable than *C*_3*v*_ symmetric one by lowering the total energy by 0.1 eV. Similar to Sc-silicene case, we could expect Jahn-Teller distortion to further stabilise Ti-silicene and Cr-silicene systems, nevertheless, the distortion for these two system turned out to be rather weak (The modification of position of all atoms is less than 0.005 *Å*, and total energy of distorted system which no longer respect the *C*_3*v*_ symmetry is lower by ~3 *m*eV).

The resulting magnetic moments and possible Jahn-Teller distortion aforementioned can be understood in the light of symmetry considerations. When TM are deposited on high symmetry sites of silicene (H, T*_A_*, T*_B_*), the 3*d* subshell of adatom split into three groups under the *C*_3*v*_ symmetric crystal field of system: the 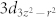 state corresponding to *A*_1_ symmetry group, the twofold degenerate *E*_1_ group consisting of the 3*d_xz_* and 3*d_yz_* states and the *E*_2_ group consisting of the 3*d_xy_* and 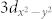 states. Therefore, the three groups of 3*d* states hybridise with *π* orbitals of silicene weakly or strongly according to the different symmetrical properties in similar way as in Benzene^38^ and graphene cases^39^. Since 3d orbitals are anti-bonding states, the energy order of them are usually 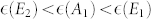 and in general 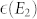 and 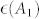 are close to each other due to similar hybridization strength with *π* orbitals. After incorporating spin polarization, the 3 groups of states split, according to different splitting energy, into 10 spin polarized orbitals. Meanwhile, the outer 4*s* electrons of adatoms experience relatively large electrostatic interaction from the *π* manifold of silicene than 3*d* shells due to its spherical symmetry and delocalized nature, making possible charge transfer from the 4*s* to 3*d* shells. Thus, the crystal field splitting, spin splitting, together with the occupation number, mostly dominate the electronic structure of adsorbed TM ions.

**Figure 2:**
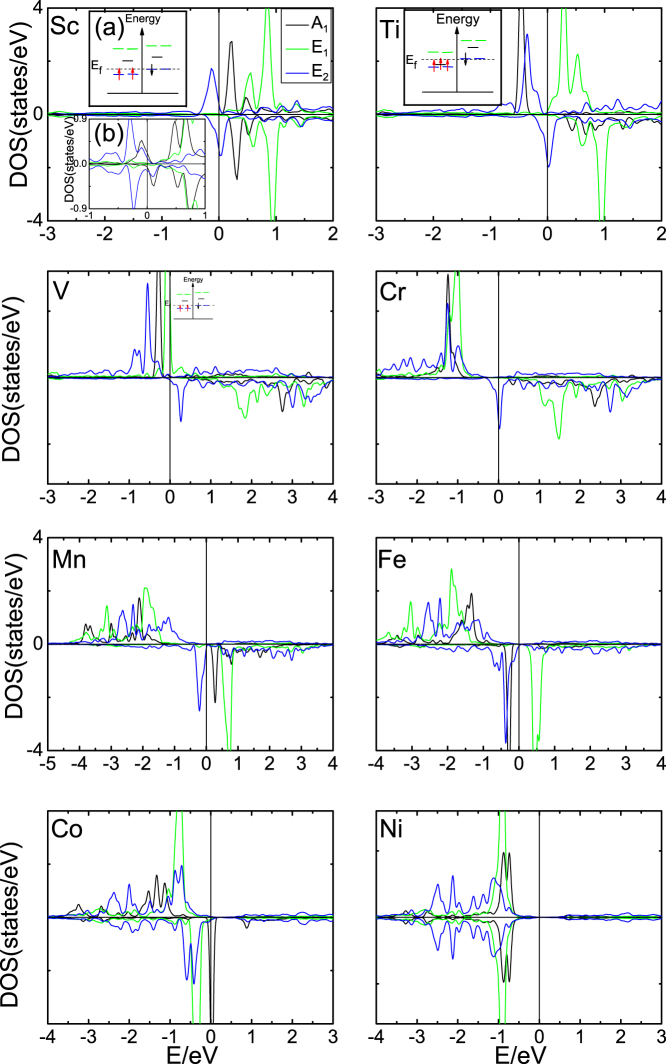
Projected density of states within generalized gradient approximation. Projected density of states (PDOS) of all 3*d* transition metals adsorbed on the stable site (Hollow) of monolayer silicene from generalized gradient approximation (GGA), where positive (negative) values are for majority (minority) spin. The inset (b) in Sc indicates the Jahn-Teller distorted PDOS. The Fermi energy is set to 0 eV.

In the case of Sc-silicene, the spin splitting for Sc is relatively weak (around 0.2 eV), which is smaller than ligand field splitting between *E*_2_ and *A*_1_, and totally there are 3 electrons occupying 3d orbitals (see Fig. 2). Therefore, two of these 3 electrons occupy the majority *E*_2_ orbitals and the other one occupies doubly degenerate minority *E*_2_ orbitals, leading to 1 *μ_B_* magnetic moment and potential Jahn-Teller distortion discussed above. For Ti-silicene, owing to the relatively large splitting of *A*_1_ (1 eV), the majority *A*_1_ orbital is occupied before the doubly degenerate minority *E*_2_ orbitals as indicated in inset of Ti's projected density of states (PDOS) from GGA (Fig. 2), resulting in peaks at Fermi level and magnetic moment of 2 *μ_B_*. For V, the spin splitting is much larger and high spin state with 5 *μ_B_* moment state is realized, which is essential to the realization of QAHE in silicene as has been discussed before. The other cases can be understood in similar arguments.

Since the strong correlation effect of 3*d* electrons is not negligible for a practical description of adsorption, we next consider GGA + *U* case. After full relaxation, the adsorption geometry of adatom-silicene system is strongly altered compared with GGA case (see Supplementary Fig. S3). And most 3*d* TM still favor H site (except Mn, which now energetically favors T*_A_* site). Clearly, the geometry change of adatom-silicene systems are the direct consequence of on-site Coulomb interactions among 3*d* electrons, which modify the electron distribution in 3*d* and 4*s* shells of adatoms and *π* orbitals of silicene as can be seen from the changes of magnetic moments (see Supplementary information for detailed discussion).

### Topological states from Chern number analysis

In this part, we turn to the main finding of this work, namely the QAHE in the absence of external field via doping 3*d* TM and the prediction of electrically tunable topological states. As has been shown in Refs. 12, 14, the QAHE could be realized via doping certain 3*d* or 5*d* adatoms on the hollow site of graphene. In Fe doped graphene case^12^, the QAHE gaps occurs around the Dirac *K* points of the Brillouin zone, and the low energy physics can be described by a Hamiltonian for graphene in the presence of extrinsic Rashba SOC (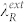) and exchange field (*M*)^40^ introduced solely by the adatom. Here in silicene, however, when depositing 3*d* TM on the stable adsorption site or applying a perpendicular external electric field, the induced inequality of AB sublattice potential (Δ) necessarily arise and compete with magnetization^26^,^27^. And the QAHE in silicene lives only in certain ranges of parameter space^26^. Moreover, owing to the low buckled structure, there exists the so called intrinsic Rashba SOC (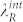)^33^. The interplay between the two kind of Rashba SOC (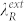 and 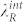) will lead to an electrically tunable topological phase transition as we will demonstrate later. Below we identify conditions for the realization of QAHE in silicene based on a effective Hamiltonian^33^ by introducing a staggered AB sublattice potential besides SOC (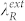 and *λ_so_*) and exchange field (*M*), which is similar to the one used in Ref. 26.

In the basis of {*A*, *B*} : {↑, ↓}, the Hamiltonian reads: 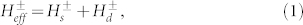
with 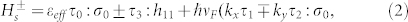

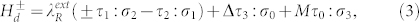

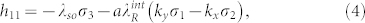
where 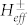 are the total Hamiltonian for the two inequivalent Dirac points *K* (+) and −*K* (−), 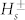 are the low energy effective Hamiltonian for the QSH insulator silicene, 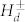 include all the effects introduced by the 3*d* dopants, including effective spin-dependent magnetic field *M*, site-dependent staggered potential Δ and the resulting extrinsic Rashba SOC 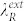. The *τ* and *σ* are the Pauli matrices acting separately on pseudospin (sublattice) and spin space, *ε_eff_* stands for the *ε*_1_ − *λ*_2*nd*_ term in Ref. 25, *ν_F_* and *a* are the Fermi velocity and the lattice constant, respectively, and *λ_so_* is the effective SOC.

**Figure 3:**
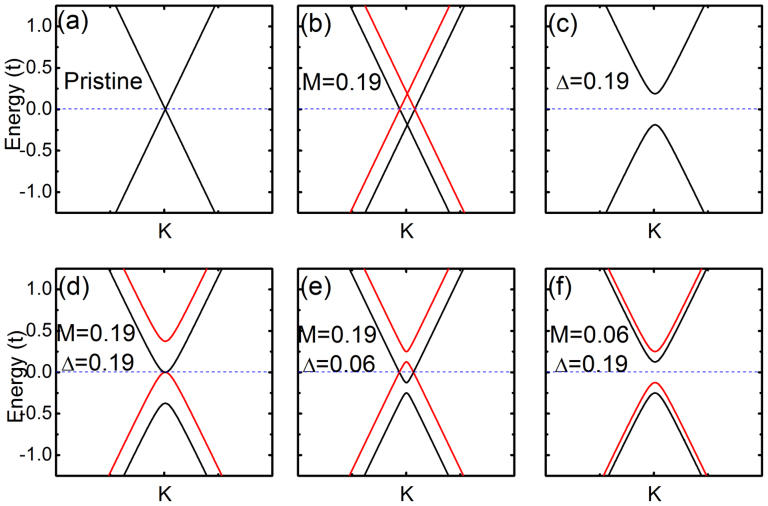
Evolution of band structure around valley *K*. The evolution of band structure around valley *K* from the interplay between exchange field *M* and staggered potential Δ (in unit of *t*). The red (black) lines are for the majority (minority) spin. (a). The band structure of pristine silicene with perfect Dirac-like energy dispersion. (b). The spin degeneracy is lifted when only exchange field *M* is turned on. (c). The system becomes insulating with the valence and conduction bands twofold degenerated when only staggered potential Δ is added. (d). When *M* = Δ, there always exists a degenerate point right at the Fermi level. (e). When *M* > Δ, the two spin subbands near Fermi level cross, resulting a circular Fermi surface. (f). When *M* < Δ, the system enters insulating state.

By diagonalizing the above Hamiltonian, the electronic structure around each valley (*K* and −*K*) in Brillouin zone can be obtained (the *ε_eff_* term can be safely ignored). From the interplay between exchange field (*M*) and staggered potential (Δ) (see Fig. 3), we conclude that energy bands with opposite spin intersect, which is essential to the QAHE in Fe doped graphene, only when *M*/Δ > 1. This is different from that in Ref. 40, where Δ = 0 and there always exists 2 degenerate points around Dirac point as long as *M* ≠ 0.

**Figure 4:**
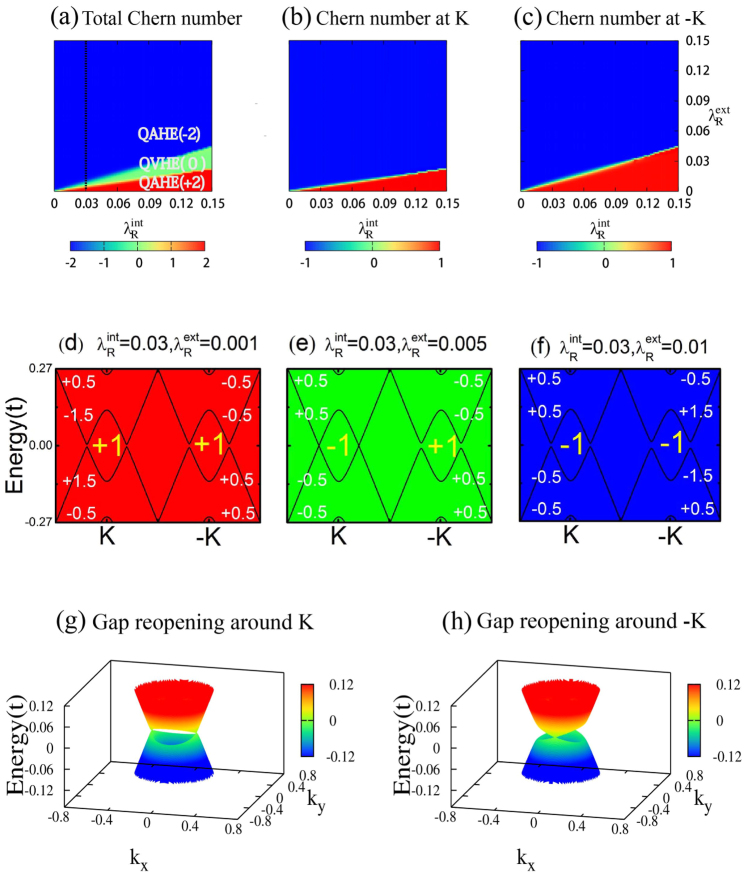
The transition of Chern number by tuning Rashba SOC. The transition of Chern number by tuning 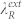 and 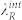 (in unit of *t*). (a) Three topological nontrivial states, QAHE(2), QVHE(0) and QAHE(−2) with Chern number +2, 0, and −2, can be obtained from different combination of 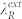 and 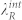. (b) and (c) represent the the variation of 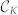 and 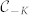. (d), (e) and (f) depict the band structure along *k_y_* = 0 line in *BZ* for the three topological states (QHE(2), QVHE(0) and QHE(−2)) in (a). The yellow integers (±1) represent 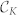 and 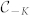, corresponding to the sum of topological charge of each valence bands (the white ±0.5 and ±1.5). (g) and (h) show the gap closing around *K* and −*K*. They are the transition states from QAHE(+2) to QVHE(0) and from QVHE(0) to QAHE(−2), respectively.

We found that either the extrinsic or the intrinsic Rashba SOC would lead to insulating state when starting from the case *M*/Δ > 1. To identify the topological properties of the resulting insulating state, we resort to the Chern number (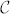) analysis^41^. The 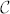 can be obtained by the integral over the first Brillouin zone (*BZ*): 
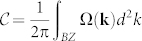
. The 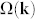 is the usual Berry curvature of all occupied states^42^: 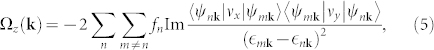
where *f_n_* is the Fermi-Dirac distribution function for band *n*, *ψ_n_*_**k**_ is the Bloch function of eigenenergy 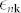, *ν_x_*, *ν_y_* are the velocity operators. And anomalous Hall conductivity is readily given by 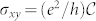. Interestingly, the extrinsic Rashba SOC (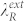) gives insulating state with 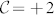 while the intrinsic one (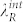) leads to that with 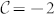. One may expect that different Chern number state can be realized if tuning the two types of Rashba SOC properly in experiments. Fig. 4(a) indicates that this is indeed the case, where 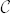 can take integer value of +2, 0, −2 with different combination of 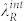 and 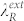. Careful study shows that the tunable 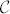 originates from different response of bulk gap to the 2 types of Rashba SOC (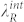 and 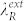) around *K* and −*K*. When increasing 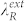 while keeping 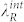 fixed, for example, at 0.03*t* (*t* = 1.6 eV is the nearest neighbor hopping parameter^33^) as shown in Fig. 4(a), we can see clearly the transition of Chern number of each valley (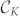 and 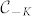) from +1 to −1 but with different rate, i.e., 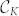 experiences a topological transition earlier than 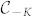 (see Figs. 4(b)–4(c)). The step change of 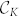 and 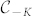 is justified by the observation of bands touching and gap reopening around each valley (Figs. 4(d)–4(f)). Notice that the rotational symmetry of the effective Hamiltonians along *z* direction in any angle is broken after bringing in the Rashba SOC terms, and hence the band touching happens only on *k_y_* = 0 line in *BZ* for valley *K* (Fig. 4(g)) while *k_x_* = 0 line for valley −*K* (Fig. 4(h)). Consequently, the system can be in QAHE phase (with 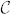 being +2 or −2) or QVHE (with 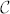 being 0 and 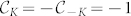) depending on different value of 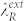, which is controllable through an external gate voltage.

The effective SOC (*λ_so_*), however, further breaks the particle-hole symmetry of the above Hamiltonian, making energy bands shift up (at valley *K*) or down (at valley −*K*) relative to the Fermi level while leaving the topological charge of each valley unchanged. Hence, as long as the shifting is small, the system is still insulating and the above discussion of topological transition remain valid.

### Verification of quantum anomalous Hall effect in 3*d* TM doped silicene

**Figure 5:**
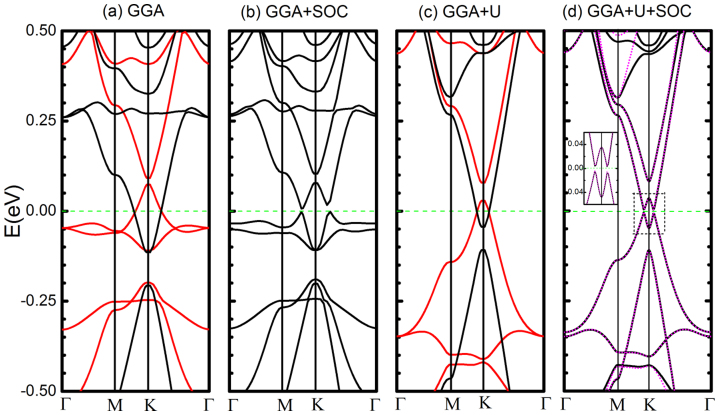
The band structures of V doped silicene. (a) The band structures of V doped silicene from GGA ((a1)–(a2)) and GGA + *U* ((a3)–(a4)), respectively. The red (black) color in (a1) and (a3) correspond to majority spin (minority spin) subbands. After including SOC effect, a gap is opened at the Fermi level ((a2) and (a4)). In (a4), the band structure from Wannier interpolation is also shown in pink dashed lines.

Some of the topologically non-trivial phases can be realized in 3*d* TM doped silicene as predicted by our first-principles calculations. For example, we notice that the opposite spin subbands cross around the Fermi level in Ti, V, Cr, Mn doped silicene due to relatively large magnetization (see Supplementary Table S1). This is closely resembling to the band inversion case (*M*/Δ > 1) as studied in TB model above (see Fig. 3(e)). Meanwhile, for these systems the spin-up and spin-down subbands near Fermi level are also gapped (Fig. 5(a) and Fig. 5(c)), which makes these systems candidates for half-metallic materials if tuning Fermi level properly. We take V-silicene as a prototype and discuss in detail, as this system is insulating with an energy gap around 6 *m*eV (Fig. 5(b)) when only the SOC is turned on. We find the SOC induced band gap in V-silicene is stable even strong correlation effect of V is considered by including effective *U* value ranging from 0 to 6 eV (Fig. 5(d)).

**Figure 6:**
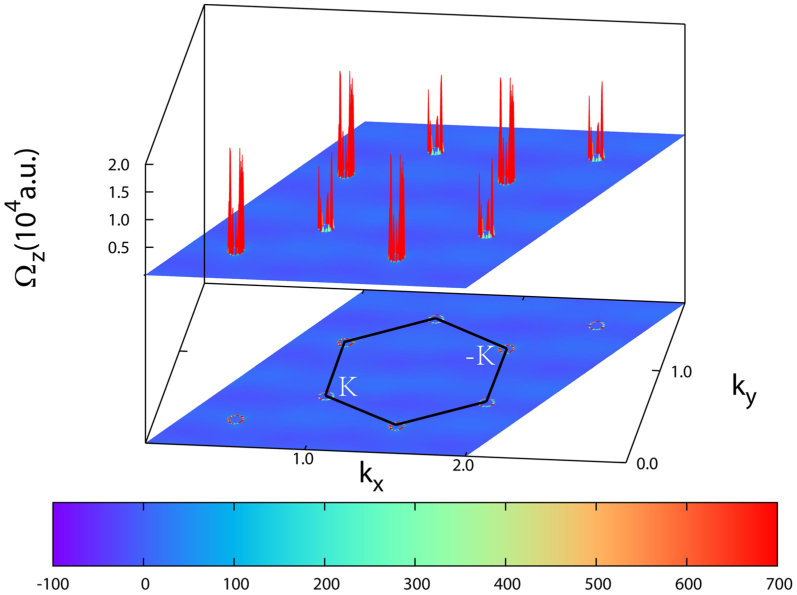
The Berry curvature distribution of V doped silicene. The distribution of Berry curvature (Ω*_z_*(*k*)) of all occupied states in V doped silicene from GGA + *U* + SOC. The first Brillouin zone is marked out with black hexagon. The small red circles in the projection drawing represent the most non-zero values of Berry curvature.

From the effective TB model analysis above, the V-silicene system could be in one of the three topologically nontrivial states (QAHE(+2), QVHE(0), QAHE(−2)) when only SOC is turned on. Thus we need obtain an accurate Berry curvature distribution and hence Chern number in first-principles level in V doped silicene^43^,^44^. As indicated in Fig. 5(d), the band structure from first principle is well reproduced by Wannier interpolation. In Fig. 6, the Berry curvature (Ω*_z_*(*k*)) in *k*-space is explicitly shown. As we may observe, the most nonzero values (positive) of Berry curvature distribute around the Dirac *K* points by forming small circles, where exactly the avoided crossing happens (Fig. 5(d)). By the integral over BZ, we indeed find the Chern number of all occupied bands equals to an integer value of +2, which signals the V doped silicene is in QAHE(+2) phase. The estimated tight-binding parameters corresponding to this case (Fig. 5(d)) are *M* = 75 meV, Δ = 21 meV, *λ_so_* = 5 meV, *λ^int^* = 8 meV, *λ^ext^* = 1.5 meV. Note that the *λ^ext^* here is induced solely by Vanadium atatom.

To induce the topological phase transition in V-silicene system, one can apply an extra external electric field to increase the 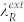. According to the phase diagram presented in Fig. 4, we can give an estimation of the magnitude of external electric field. The 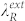 needed to enter into the QVHE(0) state is about 1.55 meV. Consequently, an extra 0.05 meV 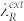 need to be supplied by external electric field *E_z_* which is about (12.5 V)/(300 nm) according to a rough estimation^33^. Further, one need apply an *E_z_* of about (225 V)/(300 nm) to generate an extra 0.9 meV 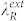 (total 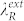 is about 2.4 meV) to enter into the QAHE(−2) phase.

## Discussion

As mentioned in former model analysis, silicene doped with certain 3d adatom could support QVHE besides the QAHE. The proposed QVHE state here in our model is interesting because it occurs in band inversion scenario, which is different from the one discussed in Ref. 45 where the spin sub-bands are not inverted. Furthermore, the QVHE here emerges from the interplay between two kind of Rashba spin-orbit couplings which is unique to silicene. We note, however, that the 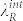 is rather small (being about 4.4 × 10^−4^ *t*)^33^. Therefore, the QVHE region in Fig. 3(a) would be quite small and it may not be easy to directly observe the QVHE from experiment. To tide it over, putting V-silicene system or its counterpart of TM doped Germanium on substrate may be a feasible solution.

To summarize, we have demonstrated that the 3*d* TM doped silicene can be intriguing materials as manifested by the induced strong magnetic moments, potential half-metallic property, and most importantly, sizable topologically non-trivial gaps. These features have also been confirmed in the presence of strong correlation effect of 3*d* TM, which is pretty important for practical implementation of these properties in silicene. Moreover, we predicted the emerging of electrically controllable topological states (QAHE and QVHE phases as characterized by different Chern number) in certain TM-silicene systems where the energy bands are inverted. Our work may provide new candidate for the realization of QAHE and platform to manipulate topological states electrically.

## Methods

The first-principles calculations are performed based on the density functional theory (DFT)^46^ with generalized gradient approximation (GGA) in the form of Perdew-Burke-Ernzerhof (PBE) functional^34^ as implemented in Vienna Ab-initio Simulation Package (VASP)^47^. The GGA +*U* method which treats the on-site repulsion interactions of 3*d* electrons in a mean field manner is used to evaluate the strong correlation effect in TM, and a typical value of *U* = 4 eV and *J* = 0.9 eV are used for all TM concerned^35^,^36^. The lattice constant *a* = 3.86 *Å* of silicene and the buckling distance *δ* = 0.44 *Å* are obtained corresponding to the global minima on the Born-Oppenheimer surface, which agree with existing theoretical data^20^,^25^. As to the structure relaxation, all atoms are allowed to relax freely along all directions and all parameters are chosen to converge the forces to less than 0.01 eV/*Å*. A vacuum space of 20 *Å* is set to prevent the interaction between silicene and its periodic images along *c*-axis. Convergence tests with respect to energy cutoff and k points sampling are performed to ensure numerical accuracy of total energy. We find an energy cutoff of 420 eV and Γ centered Monkhorst-Pack grids of 8 × 8 × 1 for k point sampling are enough to converge the difference in total energy to around 1 *m*eV.

## Author Contributions

X.L.Z. performed calculations. X.L.Z., L.F.L., W.M.L. analyzed numerical results. X.L.Z., L.F.L., W.M.L. contributed in completing the paper.

## Supplementary Material

Supplementary Information Supplementary information for ’Quantum anomalous Hall effect and tunable topological states in 3d transition metals doped silicene‚
