# Supplementary material for: Quantum Anomalous Hall Effect and Tunable Topological States in 3d Transition Metals Doped Silicene
Source: Sci Rep. 2013 Oct 9;3:2908. doi: 10.1038/srep02908 (PMC3793221; doi:10.1038/srep02908)
Supplement: Supplementary Information — for ’Quantum anomalous Hall effect and tunable topological states in 3d transition metals doped silicene‚ [file srep02908-s1.pdf]

Supplementary information for "Quantum anomalous Hall effect and tunable topological states in 3d transition metals doped silicene"

Xiao-Long Zhang⋆, Lan-Feng Liu, Wu-Ming Liu⋆

[1]*Beijing National Laboratory for Condensed Matter Physics, Institute of Physics, Chinese Academy of Sciences, Beijing 100190, China*

⋆*e-mail: xlzhang@iphy.ac.cn; wliu@iphy.ac.cn*

Impact of strong correlation effect on adsorption structure and magnetic moments

For the sake of comparison, we define bond lengths (d_{Si-TM}) as the distance between adatom and the 3 nearest neighbour Si atoms to it, adsorption height (h_{Si-TM}) as the distance between adatom and the lower sublattice (averaged) in c-axis for all 3 adsorption sites, height above Si

atom (δ_{Si-TM}) as the distance between adatom and the Si atom underneath for T$_A$ and T$_B$ sites (Fig. S1).

In the GGA case, as can be seen from Fig. S3(a), the bond lengths and adsorption height generally decrease with increasing of atomic number when TM adsorbing on H site. However, the adsorption energy doesn't follow this trend (Fig. S2), which has minimal value of 2.44 eV for Cr and maximal value of 4.75 eV for Ni. We can see from inset in Fig. S2 that the above trend of adsorption energy is correlated to the different distortion of silicene, which is energetically characterized by the distortion energy defined as $\delta E = E_{dis} - E_s$, where E_{dis} is the energy of silicene after adsorption. The distortion energy are small for V and Cr, suggesting relatively weak interactions between these adatoms and silicene, therefore, the adsorption energy decrease from V and reaches minimal value at Cr.

When turning on the strong correlation effect, the equilibrium structure of adatom-silicene systems are strongly altered compared with GGA case. As can be seen from Fig. S3(b), the d_{Si-TM}, h_{Si-TM} and δ_{Si-TM} for all adsorbates (except Ni) are increased, especially for Ti, Cr, Mn, Fe (the bond lengths for these atoms increased by ~ 0.1 $\AA$ while for others by ~ 0.05 $\AA$, and the adsorption height also showed noticeable rise for these atoms). And the H site is still favored by most 3d TM (except Mn, which energetically favors T$_A$ site).

The change of adsorption geometry of adatom-silicene system can be attributed to the direct consequence of on-site Coulomb interactions among 3d electrons. In the case of Sc-silicene, the GGA + U predicts ~ 3 μ_B magnetic moment compared with ~ 1 μ_B in GGA case

(Fig. S3). And the reason for this is the enhanced spin splitting, which results from the effective U, makes spin-up A_1 and E_2 states of Sc occupied as indicated in PDOS of Sc in Fig. S4. For Ti-silicene system, the net magnetic moment is $\sim 3\ \mu_B$ rather than $\sim 4\ \mu_B$ as one may expected, indicating the 4s shell of Ti is empty and about one electron is transferred from Ti to silicene. This is justified by the lowering of Dirac cone at K and $-K$, though slightly distorted, beneath the Fermi level, and occupied PDOS of Si atoms closest to Ti. Similar to Ti-silicene, the Cr-silicene system retained $\sim 5\ \mu_B$ magnetic moment after transferring $\sim 1e$ to silicene, which shifts the Dirac cone down by $\sim$ 0.4 eV relative to Fermi level. In the case of Mn-silicene, especially, the T_A site is favored if the strong correlation effect is turned on, which is 0.07 eV (0.13 eV) lower in total energy than H (T_A) sites. For V, Fe, Co, Ni, $+U$ mainly enhances the spin splitting while leaving the electron distribution nearly unaffected compared with GGA case, hence the same magnetic moments (Fig. S3).

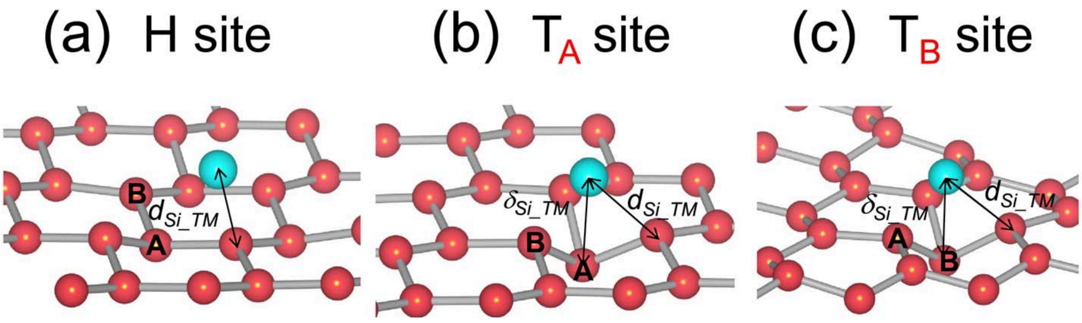

Figure S1: Schematic representation of bond parameters (d_{Si-TM} and δ_{Si-TM}) for 3 adsorption sites (a) H, (b) T$_A$, and (c) T$_B$, respectively.

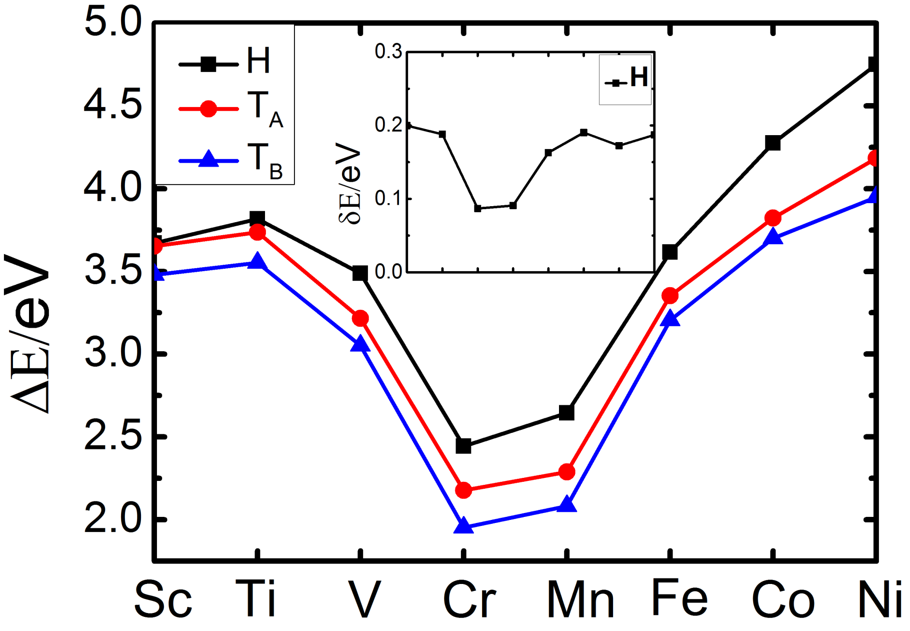

Figure S2: The adsorption energy (ΔE) of all 3d transition metals (TM) adsorbed on the three high symmetric sites of silicene monolayer, H, T$_A$ and T$_B$, in the GGA level. The inset shows the distortion energy (δE) of silicene when different TM are situated on the stable site H.

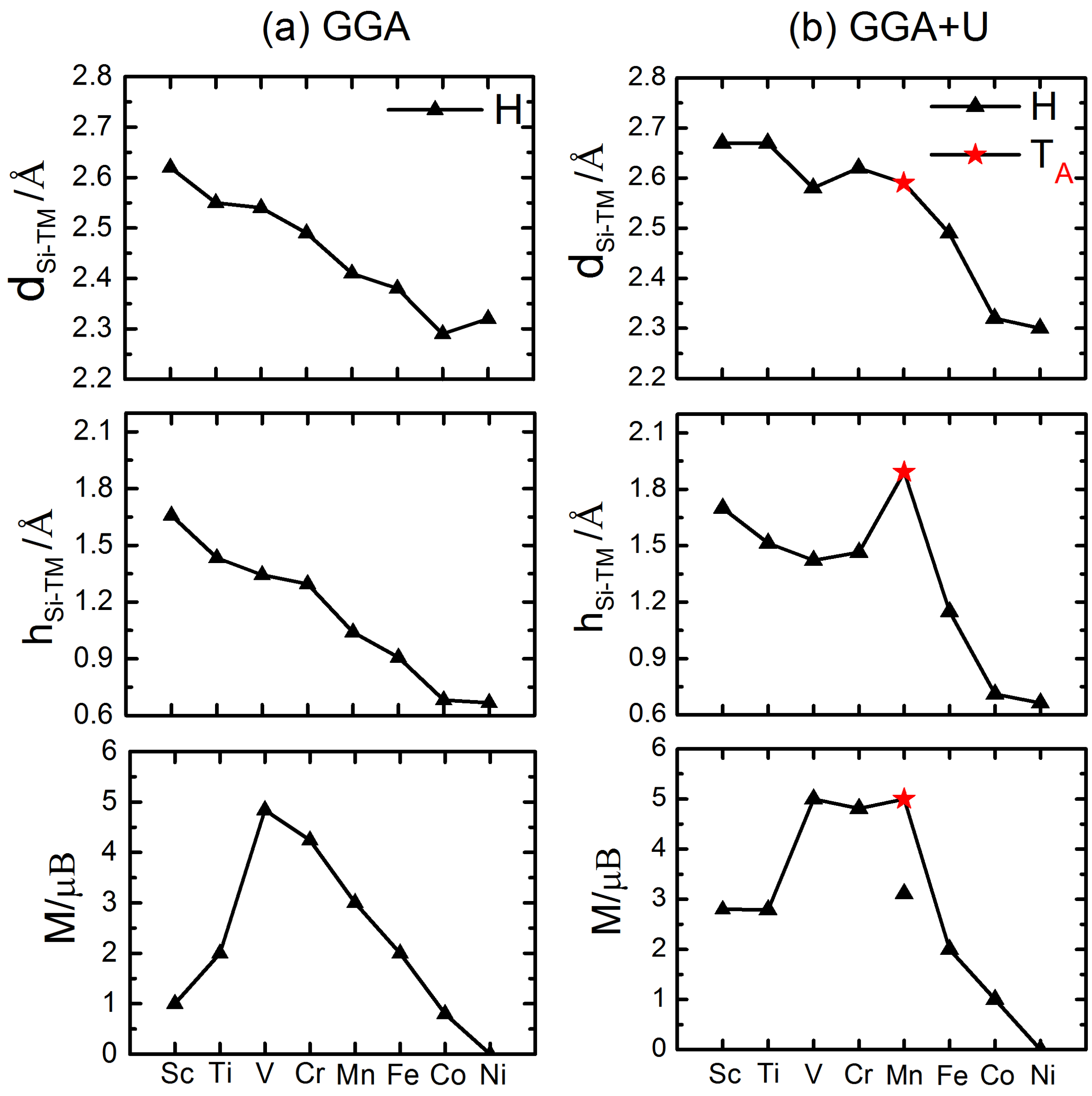

Figure S3: Structural and magnetic properties of 3d transition metals adsorbed on the Hollow site of 4×4 silicene based on (a) GGA and (b) GGA+U. For Mn in GGA+U case, the bond parameters are corresponding to the stablest T_A adsorption site (marked in red pentacle), and the magnetic moment of Hollow and T_A sites are given for comparison.

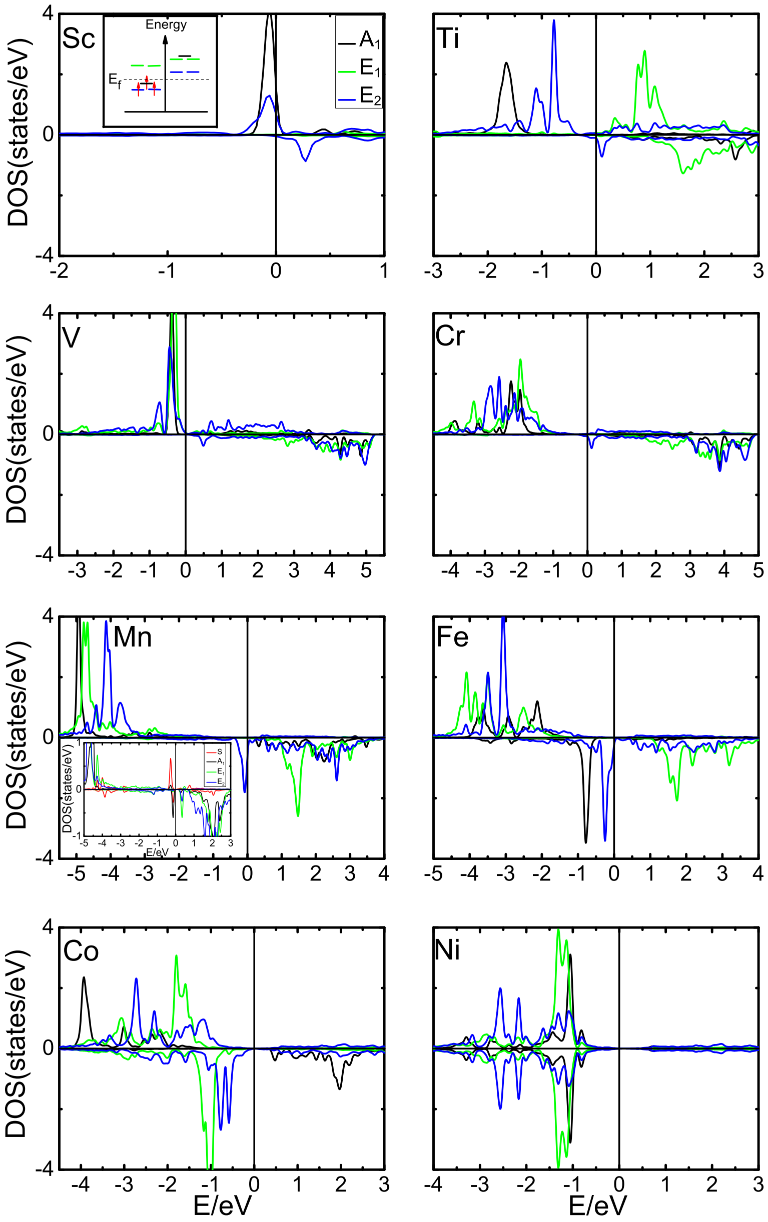

Figure S4: PDOS of all 3d transition metals adsorbed on the Hollow (H) site of monolayer silicene from GGA+U (with an effective U being 3.1eV), where positive (negative) values are for majority (minority) spin. For Mn, the PDOS including 4s orbitals are given in inset when absorbing on the stable site T_A. The Fermi energy is set to 0 eV.

Adatoms	Sc	Ti	V	Cr	Mn	Fe	Co	Ni
M (meV)	37	115	135 (75)	94	70	24	12	0
Δ (meV)	70	65	42 (21)	50	44	35	16	3

Table S1: The estimated values of M (magnetization) and Δ (staggered potential) in tight binding model for Generalized Gradient Approximation (GGA) case. For Vanadium doping, the values in parentheses are for the GGA+U case. Note that for Ti, V, Cr, Mn, M is greater than Δ.
